# Preterm Birth and Small-for-Gestational Age Neonates among Prepregnancy Underweight Women: A Case-Controlled Study

**DOI:** 10.3390/jcm10245733

**Published:** 2021-12-07

**Authors:** Emelyne Lefizelier, Emilie Misbert, Marion Brooks, Aurélie Le Thuaut, Norbert Winer, Guillaume Ducarme

**Affiliations:** 1 Department of Obstetrics and Gynaecology, Centre Hospitalier Departemental, 85000 La Roche sur Yon, France; emelyne.lefizelier@ght85.fr (E.L.); marion.brooks@ght85.fr (M.B.); 2 Department of Obstetrics and Gynaecology, Nantes University Hospital, 44000 Nantes, France; emilie.misbert@chu-nantes.fr (E.M.); norbert.winer@chu-nantes.fr (N.W.); 3 Plateforme de Méthodologie et Biostatistique, CHU Nantes, 44093 Nantes, France; aurelie.lethuaut@chu-nantes.fr

**Keywords:** pregnancy, preterm birth, underweight, small for gestational age

## Abstract

The aim of our study was to investigate whether prepregnancy underweight body mass index (BMI) is associated with preterm birth (PTB) and small-for-gestational age (SGA). This retrospective case-control study included 814 women with live singleton fetuses in vertex presentation that gave birth between January 2016 and November 2016 in two tertiary care hospitals. The study group (*n* = 407) comprised all women whose prepregnancy BMI was underweight (<18.5 kg/m^2^) and who delivered during the study period. A control group (*n* = 407) was established with women whose prepregnancy BMI was normal (18.5–24.9 kg/m^2^) by matching age and parity. Univariate and multivariate analyses were performed to compare PTB and SGA associated with prepregnancy underweight BMI. Compared with the control group, the study group had higher rates of overall PTB (10.1% vs. 5.7%, *p* = 0.02), iatrogenic PTB (4.2% vs. 1.5%, *p* = 0.02), and SGA (22.1% vs. 11.1%, *p* < 0.001). In a multivariable analysis, prepregnancy underweight BMI was associated with PTB (aOR 2.32, 95% CI 1.12–4.81) and with SGA (aOR 2.38, 95% CI 1.58–3.58). In singleton pregnancies, women’s prepregnancy underweight compared with normal BMI was associated with an increase in PTB and in SGA neonates. Identifying this specific high-risk group is pragmatic and practical for all physicians, and they should be aware about perinatal outcome among underweight women.

## 1. Introduction

Despite the current obesity epidemic, prepregnancy underweight, defined by the World Health Organization International Classification as a Body Mass Index (BMI) lower than 18.5 kg/m^2^ [1], remains a common occurrence. Maternal underweight was reported among 7.5% of pregnant women [2,3].The analysis of the literature on maternal and perinatal outcomes among underweight women shows discrepancies. In a large population-based cohort from California, no association was reported between underweight BMI and spontaneous preterm birth (PTB; <37 weeks’ gestation), due to major influences by race/ethnicity, gestational age, and parity [4].Conversely, in a retrospective cohort in Colorado, underweight women were significantly more likely to have a PTB (adjusted odds ratio 2.4; 95% confidence interval (CI) 1.4–4.2; *p* = 0.003) [5]. More recently, a meta-analysis, involving 78 studies, 1,025,794 women in developed and in developing countries, reported that prepregnancy underweight was significantly associated with PTB, compared to a normal prepregnancy BMI (adjusted relative risk (RR) 1.29, 95% CI 1.15–1.46) [6]. In the most recent retrospective cohort study in California including 950,356 California deliveries in 2007–2010 with 72,686 (7.6%) underweight women, increasing severity of prepregnancy underweight BMI was associated with increasing risk-adjusted PTB [3]. The meta-analysis also showed that underweight women had an increased risk of low-birth weight infant and small-for-gestational age (SGA), compared to a normal prepregnancy BMI (adjusted RR 1.64, 95% CI 1.38–1.94) [6].

In a retrospective case-control study, we aimed to compare PTB and SGA for underweight and normal BMI women, and to analyse the risk factors for PTB and SGA in underweight women.

## 2. Materials and Methods

### 2.1. Patient Selection

This is a retrospective case–control study. The study group comprised all women with a prepregnancy underweight BMI (<18.5 kg/m^2^), carrying a live singleton fetus in vertex presentation, who gave birth from January 2016 to November 2016 at two tertiary care hospitals. A control group of pregnant women who gave birth during the same study period with normal prepregnancy BMI (18.5–24.9 kg/m^2^) was established by matching, one-to-one, according to maternal prepregnancy age (less than 20 years, between 20 and 39 years, and over 39 years), parity (nulliparous or multiparous), month of birth, and hospital. We excluded women without first-trimester ultrasound gestational age dating, multiple pregnancy, and women with medical-indicated second trimester termination of pregnancy, intra uterine death or fetal loss before 22 weeks.

This present study was conducted in accordance with the French approved guidelines. All participants received written information. Written consent was not required for retrospective study according to the French law, but each woman got the opportunity to opt out of the analysis. The study protocol was approved by a Research Ethics Committee (Groupe Nantais d’Ethique dans le Domaine de la Santé (GNED)) on 27 June 2017 before the beginning of the study.

### 2.2. Data Collection

Maternal demographic characteristics, information regarding pregnancy follow-up and standard perinatal outcomes were collected retrospectively by one obstetrician (E.L.) from the electronic medical record database of the two hospitals included in our study. Maternal characteristics collected included maternal prepregnancy age, BMI (calculated as weight (kg)/[height (m)]^2^), based on height and the first weight noted in the obstetric record), geographic origin (North Africa, Sub-Saharan Africa, Hispanic, Asian, Overseas departments, Caucasian), severity of maternal underweight BMI (severe (BMI, <16 kg/m^2^), moderate (BMI, 16–16.99 kg/m^2^), and mild (BMI, 17–18.49 kg/m^2^)) [1], parity, smoking (defined by smoking regardless of the amount cigarettes smoked), previous uterine scar (previous caesarean section or myomectomy), previous hypertension [7], pregestational diabetes mellitus, history of depression, and history of eating behaviour disorders (anorexia or bulimia).

Pregnancy and labour characteristics collected included assisted reproductive technology-conceived pregnancy, gestational weight gain (GWG) [8], gestational diabetes mellitus (GDM), pregnancy-associated hypertensive disorders, anaemia (defined by haemoglobin < 11 g/dL before delivery) [9], intrahepatic cholestasis of pregnancy (ICP), threatened preterm labour required hospitalization, and antenatal suspicion of SGA. Maternal gestational weight gain (GWG) was calculated as measured weight at the end of pregnancy minus pre-pregnancy weight. Adequate GWG was based on the Institute of Medicine’s GWG by maternal pre-pregnancy BMI recommendations [8]. GDM was diagnosed as usual, according to international guidelines for pregnant women [10]. Women who were controlled with antenatal insulin therapy (AIT) were allowed to await spontaneous labour until 41 weeks. Women who were not controlled on diet alone or with AIT or who presented with an estimated foetal weight > 97th centile at 37 weeks were advised to undergo induction of labour at 39 weeks [11]. Pregnancy-associated hypertension disorders were determined by hypertension without proteinuria or preeclampsia (hypertension and proteinuria) after 20 weeks’ gestation in a previously normotensive woman [12]. In case of non-severe preeclampsia beyond 36 weeks, elective delivery must be considered, and in case of severe preeclampsia beyond 34 weeks, elective delivery is indicated. Intrahepatic cholestasis of pregnancy is a cholestatic disorder characterized by pruritus with onset in the second or third trimester of pregnancy and elevated serum aminotransferases and bile acid levels. According to our institutional guidelines, women who were not clinically and/or biologically controlled with ursodeoxycholic acid were advised to undergo induction of labour at 37 weeks of gestation. Antenatal suspicion of SGA was defined as an ultrasonographic estimated foetal weight < 10th centile adjusting for gestational age and sex [13]. According to French guidelines, elective delivery was recommended between 30–32 weeks and 38–39 weeks of gestation according to estimated fetal weight (less than the 3rd percentile, or between the 3rd and 10th percentile) and umbilical artery Doppler waveform (normal/absent/reversed end-diastolic velocity) [14].

Intrapartum variables collected included gestational age at delivery [15], spontaneous or iatrogenic PTB (defined as delivery less than 37 completed weeks of gestation, but analytically defined more narrowly, less than 28 weeks, 28–32 weeks, or 32–36 weeks (compared with 37–41 weeks)), type of labour (spontaneous or induced, planned caesarean delivery), mode of delivery (spontaneous or operative vaginal delivery, or caesarean section during labour), third or fourth degree perineal tears [16], postpartum haemorrhage (PPH, defined as bleeding 500 mL or greater after vaginal delivery) and severe PPH (defined as bleeding 1000 mL or greater) [17], and birth weight (calculated in grams, centile and Z-score adjusting for gestational age and the offspring’s sex) [18], Low-birth weight (LBW) neonate was defined as less than 2500 g, and macrosomia was defined as greater than 4000 g. SGA neonate was defined as birth weight less than the 10th percentile adjusting for gestational age and the offspring’s sex, and large for gestational age (LGA) neonate as birth weight greater than the 90th percentile adjusting for gestational age and the offspring’s sex [19].

In addition, we routinely measured newborns’ umbilical arterial blood gases at birth. A paediatrician examined the newborn in all cases after delivery. Infants in need of close monitoring were transferred to the neonatal intensive care unit (NICU). Immediate neonatal morbidity data recorded were 5-min Apgar score, cord pH, any transfer to the NICU, respiratory distress syndrome, neonatal hyperbilirubinemia, neonatal hypoglycaemia, intraventricular haemorrhage greater than grade 2, need for resuscitation or intubation, sepsis, seizures, and neonatal death. Respiratory distress syndrome was defined by the presence of respiratory distress, an increased oxygen requirement (FiO_2_ ≥ 0.4) and compatible chest radiographic findings without any evidence of another cause of respiratory distress [20]. Neonatal hypoglycaemia was defined as blood glucose < 40 mg/dL in the first 24 h post-delivery or blood glucose < 50 mg/dL from the second day of life [21]. Neonatal hyperbilirubinemia was recorded when the infant was treated with phototherapy after birth or admission at the neonatology department for this reason. Neonatal sepsis was defined as confirmed clinical infection with positive bacteriological tests [22].

To calculate sample size, we assumed a maximum rate of PTB of 12% among underweight women [3,6], and a minimum rate of PTB of 6% in women with a normal BMI [3,6,23,24,25]. For a power of 85% to detect a reduction in PTB from 12% to 6% between the two groups using a two-tailed t-test and alpha error of 0.05, 814 women (407 women per group) were required in the study.

### 2.3. Statistical Analysis

Continuous data were described by their means ± standard deviations and compared by t tests (or Mann-Whitney tests when appropriate), and categorical data were described by percentages and compared by χ^2^ tests (or Fisher’s exact tests when appropriate). We compared maternal and perinatal outcomes according to BMI, and specifically studied the association (assessed by multivariate logistic regression analyses) between PTB and SGA with prepregnancy underweight BMI, compared with normal BMI. If the association between PTB and SGA and a maternal characteristic was a clinically relevant potential for confounding or was already known from the literature for being linked to PTB and to SGA [3,6], the factor was added in the multivariable analyses. SAS 9.4 software (SAS Institute, Cary, NC, USA) was used was used for all analyses. *p* values < 0.05 were considered to be statistically significant.

## 3. Results

**Figure 1:**
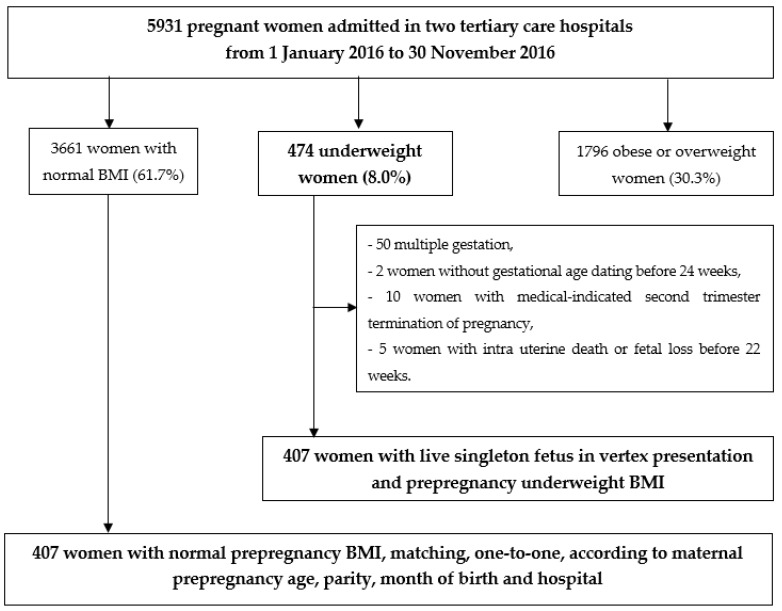
Study population.

In the analytic dataset of 5931 live births during the study period, 407 women (6.86%) presented prepregnancy underweight BMI. These women (study group) were compared to 407 matched women in a control group (Figure 1). Table 1 details the maternal and labour characteristics and maternal and neonatal outcomes. Smoking, history of depression and history of eating behaviour disorder were significantly more frequent in underweight women, (30.0% compared to 19.9%, *p* = 0.001; 6.9% compared to 3.0%, *p* = 0.01; and 6.1% compared to 2.5%, *p* = 0.01, respectively). GWG was significantly higher in study group (13.1 ± 4.2 compared to 12.3 ± 4.7 kg, *p* = 0.02). Threatened preterm labour requiring hospitalization was twice as frequent in cases (8.6% compared to 3.7%, *p* < 0.01), and ICP was four-times as frequent in control group (0.5% compared to 2.2%, *p* = 0.03). No difference was observed between groups regarding geographic origin (Table 1).

Gestational age at delivery was significantly different, but not clinically meaningful, in both groups (39.2 ± 2.2 in study group compared to 39.6 ± 1.7 weeks in control group, *p* = 0.001), and the rates of overall PTB and iatrogenic PTB were higher in the study group (10.1% compared to 5.7%, *p* = 0.02; and 4.2% compared to 1.5%, *p* = 0.02, respectively) (Table 1). The groups differed according to smoking, inadequate GWG, and ICP (Table 2). More than half iatrogenic PTB (9/17) was due to antenatal suspicion of SGA in underweight women, compared to a third in the control group (2/6).

In the multivariable logistic regression analysis adjusted for potential confounders (maternal age, parity, smoking, inadequate GWG, ICP, GDM, pregnancy-associated hypertensive disorders, and antenatal suspicion of SGA), maternal prepregnancy underweight (compared with normal) BMI was significantly associated with PTB (adjusted odds ratio (aOR) 2.32, 95% confidence interval (CI) 1.12–4.81; *p* = 0.02) (Table 3).

Smoking (aOR 3.01, 95% CI 1.45–6.25), inadequate GWG (aOR 4.48, 95% CI 2.04–9.82), antenatal suspicion of SGA (adjusted OR 36.44, 95% CI 8.95–148.30), and ICP (aOR 28.46, 95% CI 5.91–137.13) were also significantly associated with PTB (Table 3).

Increasing rate of PTB was observed with accordance to increasing severity of underweight (7.8% in mild, 14.1% in moderate and 42.2% in severe underweight) (Table 2). Maternal severe thinness (BMI < 16 kg/m^2^) (compared with mild thinness (BMI, 17–18.49 kg/m^2^)) was significantly associated with PTB (aOR 2.33, 95% CI 1.18–19.35) in the multivariable logistic regression analysis adjusted for potential confounders (Table 3).

Birth weight (3055 ± 580 compared to 3280 ± 511 g, *p* < 0.001) and birth weight Z-score (−0.2 ± 1.0 compared to 0.14 ± 1.0, *p* < 0.001) were significantly lower for the newborns of those in the maternal underweight compared with the control women. The proportions of SGA and LBW neonates were significantly higher for the study group than the control group (22.9% compared to 11.1%, *p* < 0.001, and 14.3% compared to 5.9%, *p* < 0.001). Immediate neonatal morbidity was similar in both groups (Table 1). The groups differed according to maternal age, smoking, history of depression, history of eating behaviour disorders, inadequate GWG, GDM and pregnancy-associated hypertensive disorders (Table 4).

In the multivariable logistic regression analysis adjusted for potential confounders, only maternal underweight BMI was significantly associated with SGA (aOR 2.38, 95% CI 1.58–3.58; *p* < 0.001).

## 4. Discussion

This study reports a retrospective case-control analysis of women who prepregnancy BMI was underweight (study group) or normal (control group by matching age and parity), and PTB and SGA according to prepregnancy BMI. We found that maternal prepregnancy underweight BMI was associated with higher rates of PTB and SGA than normal BMI. Increasing rate of PTB was observed in accordance with increasing severity of underweight BMI.

Our data are robust. We included women who gave birth at two tertiary care hospitals that are in the same perinatal network with common guidelines concerning pregnancy and delivery management. We included women with normal BMI who were matched for the major factors known to affect pregnancy outcomes (maternal age and parity). All neonates were also routinely examined by a qualified neonatologist after delivery. Moreover, history of eating behaviour disorder and history of depression, that are well-known to be associated with PTB and SGA in underweight women, were systematically sought in medical files [26,27].

Our results must be interpreted in light of certain limitations. First, the main limitation of this study is the retrospective design which is a source of bias inherent to such investigations. Prepregnancy BMI was based on height and the first weight noted in the obstetric record. This first weight noted in the electronic medical record database of the two hospitals corresponded to the statements of the woman at the first exam during pregnancy that may represent a memory bias. Nevertheless, some of these women were seen in hospitals before pregnancy, and prepregnancy weight was well-notified in the electronic record. Second, although the sample size of this retrospective case–control study was large (*n* = 814), our study may lack sufficient statistical power to detect small but clinically relevant differences in infrequent perinatal outcomes. Moreover, women in the control group were matched by the major factors known to affect pregnancy outcomes (maternal age and parity); however, we could not exclude the possibility that additional hidden confounders that were unfortunately not recorded (ie, socio-economic status, level of education, and haemoglobin level at the beginning of the pregnancy) could explain the differences observed between groups and may represent reported bias for rates of PTB and SGA [28,29]. We also considered that using matching, one-to-one, according to month of birth and hospital especially avoided significant variation regarding diagnosis of PTB and SGA, and need for obstetric intervention. These limitations notwithstanding, our study supports the continued close multidisciplinary follow-up of underweight women during pregnancy to prevent PTB and SGA with regular evaluation of cervix status and uterine contractions and regular ultrasound fetal growth monitoring during third trimester. Identifying these specific high-risk pregnant women is pragmatic and practical for all physicians, this information could be used for prepregnancy counselling in these women with low BMI, and physicians should be aware of perinatal outcome among underweight women.

Rates of underweight pregnant women (6.9%), overall PTB (10.1%) and medically induced PTB (4.2%) were observed in accordance with previous publications [2,4,30]. Several studies have evaluated the association between maternal underweight BMI and PTB [3,4,5,6,31]. The largest meta-analysis, involving 78 studies and 1,025,794 women, reported that prepregnancy underweight was significantly associated with PTB, compared to a normal prepregnancy BMI (adjusted relative risk (RR) 1.29, 95% CI 1.15–1.46) [6]. However, this meta-analysis included a potential major bias inherent in combining studies from developed and developing countries [6]. Our results are consistent with other well-established findings in the literature: increasing severity of prepregnancy underweight BMI was associated with increasing risk-adjusted PTB [3,24,30], maternal underweight BMI was also associated with moderate PTB (between 32 and 36 weeks) rather than with severe PTB (less than 28 weeks) [30,31], and iatrogenic PTB was higher among underweight women [4]. In agreement with the literature [6,23,24,25,32], our study also showed that more than half iatrogenic PTB (9/17) was due to antenatal suspicion of SGA in underweight women, compared to a third in the control group. Despite the small sample size of the study, antenatal suspicion of altered fetal growth seems to be an important risk factor for PTB.

## 5. Conclusions

Our study showed that women’s prepregnancy underweight, compared to women with normal BMI, was associated with an increase in PTB and in SGA neonates in singleton pregnancies. This study might be useful for prepregnancy counselling in women with low BMI who are willing to get pregnant.

## Figures and Tables

**Table 1:** Maternal and labour characteristics and maternal and neonatal outcomes according to the prepregnancy BMI.

	Underweight BMI (<18.5 kg/m^2^) *n* = 407	Normal BMI (18.5–24.9 kg/m^2^) *n* = 407	*p*-Value
**Maternal characteristics**			
Maternal age (years)			
Less than 20 years	18 (4.4)	18 (4.4)	-
Between 20 and 39 years	376 (92.4)	376 (92.4)	-
Over 39 years	13 (3.2)	13 (3.2)	-
BMI before pregnancy (kg/m^2^)	17.5 ± 0.9	21.5 ± 1.8	<0.001
Nulliparity	165 (40.5)	165 (40.5)	-
Geographic origin			
Caucasian	376 (92.4)	366 (89.9)	0.22
North Africa	11 (2.7)	15 (3.7)	0.43
Sub-Saharan Africa	11 (2.7)	11 (2.7)	>0.99
Asian	6 (1.5)	9 (2.2)	0.45
Overseas Departments	3 (0.7)	6 (1.5)	0.34
Hispanic	0	0	-
Smoking	122 (30.0)	81 (19.9)	0.001
Previous caesarean delivery	44 (10.8)	31 (7.6)	0.12
Previous hypertension	3 (0.7)	0	0.25
Pregestational diabetes mellitus	3 (0.7)	5 (1.2)	0.22
History of depression	28 (6.9)	12 (3.0)	0.01
History of eating behaviour disorder	25 (6.1)	10 (2.5)	0.01
ART-conceived pregnancy	26 (6.4)	24 (5.9)	0.77
Gestational weight gain (kg)	13.1 ± 4.2	12.3 ± 4.7	0.02
Inadequate gestational weight gain	179 (46.4)	174 (44.7)	0.65
Gestational diabetes mellitus	29 (7.1)	25 (6.1)	0.57
Intrahepatic cholestasis of pregnancy	2 (0.5)	9 (2.2)	0.03
Anaemia	198 (48.8)	190 (47.2)	0.64
Threatened preterm labour	35 (8.6)	15 (3.7)	<0.01
Pregnancy-associated hypertensive disorders	4 (1.0)	6 (1.5)	0.52
Antenatal suspicion of SGA *	13 (3.2)	5 (1.2)	0.06
**Labour characteristics**			
Gestational age at delivery (w)	39.2 ± 2.2	39.6 ± 1.7	0.001
Preterm birth	41 (10.1)	23 (5.7)	0.02
Less than 28 weeks	2 (0.5)	1 (0.3)	
28 to less than 32	5 (1.2)	2 (0.5)	
32 to less than 36	34 (8.4)	20 (4.9)	
Iatrogenic preterm birth	17 (4.2)	6 (1.5)	0.02
Planned caesarean delivery	22 (5.4)	19 (4.7)	0.64
Induced labour	65 (16.0)	78 (19.2)	0.23
Spontaneous vaginal delivery	316 (77.6)	309 (75.9)	0.56
Operative vaginal delivery	47 (11.6)	51 (12.5)	0.67
Caesarean section during labour	44 (10.8)	47 (11.6)	0.74
**Maternal outcome**			
3rd or 4th-degree perineal lacerations	4 (1.1)	3 (0.8)	0.53
Postpartum haemorrhage (PPH)	30 (7.6)	33 (8.2)	0.50
Severe PPH (blood loss > 1000 mL)	12 (3.1)	16 (4.0)	0.47
**Neonatal outcome**			
Birth weight (g)	3055 ± 580	3281 ± 511	<0.001
Birth weight (centile)	35 ± 27	46 ± 27	<0.001
Birth weight Z-score	−0.2 ± 1.0	0.14 ± 1.0	<0.001
SGA ^†^	93 (22.9)	45 (11.1)	<0.001
Birth weight 2500 g or less	58 (14.3)	24 (5.9)	0.001
LGA ^‡^	9 (2.2)	26 (6.4)	<0.01
Birth weight 4000 g or more	13 (3.2)	27 (6.6)	0.02
5-min Apgar score less than 7	5 (1.2)	3 (0.7)	0.73
pH < 7.10	8 (2.0)	20 (5.3)	0.02
Transfer to NICU	53 (13.0)	44 (10.8)	0.33
NICU hospitalization longer than 24 h	26 (6.4)	21 (5.2)	0.45
Respiratory distress syndrome	29 (7.1)	33 (8.1)	0.60
Neonatal hyperbilirubinemia	17 (4.2)	6 (1.5)	0.02
Neonatal hypoglycaemia	7 (1.7)	3 (0.7)	0.20
Intraventricular haemorrhage greater than grade 2	0	3 (0.7)	0.25
Need for resuscitation or intubation	3 (0.7)	2 (0.5)	0.98
Sepsis	4 (1.0)	5 (1.2)	0.96
Seizures	0	1 (0.3)	0.87
Neonatal death	1 (0.3)	3 (0.7)	0.62

ART, assisted reproductive technologies; SGA: small-for-gestational age; LGA: large-for-gestational-age; NICU, neonatal intensive care unit. * Antenatal suspicion of SGA: ultrasonographic estimated foetal weight < 10th centile adjusting for gestational age and sex [14]. † SGA: birth weight < 10th centile adjusting for gestational age and the offspring’s sex [21]. ‡ LGA: birth weight < 90th centile adjusting for gestational age and the offspring’s sex [21]. Continuous data are expressed as means ± standard deviations; discrete data are expressed as *n* or *n* (%). Student t test, χ^2^ test, non-parametric Mann-Whitney test, and Fisher’s exact test were used as appropriate. A *p*-value of 0.05 was considered significant.

**Table 2:** Univariate analysis of preterm birth among underweight and normal prepregnancy BMI women.

	Preterm Birth *		
	No (*n* = 750)	Yes (*n* = 64)	*p*
Underweight women (BMI, <18.5 kg/m^2^)	366 (48.8)	41 (64.1)	0.02
Categories of underweight women			0.02
Severe thinness (BMI, <16 kg/m^2^)	277 (36.9)	27 (42.2)	
Moderate thinness (BMI, 16–16.99 kg/m^2^)	69 (9.2)	9 (14.1)	
Mild thinness (BMI, 17–18.49 kg/m^2^)	20 (2.7)	5 (7.8)	
Normal prepregnancy BMI women (BMI, >18 kg/m^2^)	384 (51.2)	23 (36.0)	0.02
Maternal age 40 years or greater	25 (3.3)	1 (1.6)	0.45
Nulliparity	302 (40.3)	28 (43.8)	0.59
Smoking	173 (23.1)	30 (46.9)	<0.001
ART-conceived pregnancy	22 (2.9)	2 (3.1)	0.93
Inadequate GWG	322 (44.2)	31 (67.4)	<0.01
Pregnancy-associated hypertensive disorders	13 (1.8)	3 (4.7)	0.15
Threatened preterm labour required hospitalization	25 (3.3)	25 (39.1)	<0.001
GDM	45 (6.0)	9 (14.1)	0.02
Antenatal suspicion of SGA	36 (4.8)	14 (21.9)	<0.001
Anaemia	360 (48.3)	28 (44.4)	0.56
ICP	5 (0.7)	6 (9.4)	<0.001

BMI, body mass index; ART, assisted reproductive technology; GWG, gestational weight gain; GDM, gestational diabetes mellitus; ICP, intrahepatic cholestasis of pregnancy. Data are mean ± standard deviation or *n* (%) unless otherwise specified. χ^2^ test and Fisher’s exact test were used as appropriate. A *p* value of 0.05 was considered significant. * Preterm birth was defined as delivery less than 37 completed weeks of gestation.

**Table 3:** Multivariate analysis of preterm birth among underweight and normal prepregnancy BMI women.

	Preterm Birth (*n* = 64)	
Variable *	Adjusted OR (95% CI) *	*p*-Value
Underweight women (BMI less than 18.5 kg/m^2^)	2.32 (1.12–4.81)	0.02
Maternal age 40 years or greater	0.40 (0.04–3.48)	0.06
Nulliparity	1.78 (0.87–3.61)	0.11
Smoking	3.01 (1.45–6.25)	0.01
Inadequate GWG	4.48 (2.04–9.82)	0.001
Pregnancy-associated hypertensive disorders	7.26 (0.71–74.42)	0.09
GDM	2.00 (0.72–5.60)	0.19
Antenatal suspicion of SGA	36.44 (8.95–148.30)	<0.001
ICP	28.46 (5.91–137.13)	<0.001

OR, odds ratio; CI, confidence interval; BMI, body mass index; GWG, gestational weight gain; GDM, gestational diabetes mellitus; ICP, intrahepatic cholestasis of pregnancy. * Adjusted for maternal age, parity, inadequate GWG, ICP, GDM, pregnancy-associated hypertensive disorders, and antenatal suspicion of SGA.

**Table 4:** Univariate analysis of SGA neonates among underweight and normal prepregnancy BMI women.

	SGA Neonates *		
	No (*n* = 676)	Yes (*n* = 138)	*p*
Underweight women (BMI, <18.5 kg/m^2^)	314 (46.5)	93 (67.4)	<0.001
Categories of underweight women			<0.0001
Severe thinness (BMI, <16 kg/m^2^)	16 (2.4)	9 (6.5)	
Moderate thinness (BMI, 16–16.99 kg/m^2^)	56 (8.3)	22 (15.9)	
Mild thinness (BMI, 17–18.49 kg/m^2^)	242 (35.8)	62 (44.9)	
Normal prepregnancy BMI women (BMI, >18 kg/m^2^)	362 (53.5)	45 (32.6)	<0.001
Maternal age 40 years or greater	19 (2.8)	7 (5.1)	0.17
Nulliparity	266 (39.4)	64 (46.4)	0.13
Smoking	145 (21.5)	58 (42.0)	<0.001
History of eating behavior disorders	24 (3.6)	11 (8.0)	0.02
History of depression	25 (3.7)	15 (10.9)	<0.001
ART-conceived pregnancy	41 (6.1)	9 (6.5)	0.84
Inadequate GWG	274 (42.7)	79 (59.4)	<0.001
Pregnancy-associated hypertensive disorders	7 (1.0)	3 (2.2)	0.28
GDM	48 (7.1)	6 (4.4)	0.24
Anaemia	331 (49.3)	57 (41.6)	0.10
ICP	11 (1.6)	0	0.99

BMI, body mass index; ART, assisted reproductive technology; GWG, gestational weight gain; GDM, gestational diabetes mellitus; ICP, intrahepatic cholestasis of pregnancy. Data are mean ± standard deviation or *n* (%) unless otherwise specified. χ2 test and Fisher’s exact test were used as appropriate. A *p* value of 0.05 was considered significant. * SGA was defined as birth weight less than the 10th percentile adjusting for gestational age and the offspring’s sex [21].

## Data Availability

The data presented in this study are available on request from the corresponding author. The data are not publicly available due to institutional policy.
